# Constructing Asymmetric Polyion Complex Vesicles via Template Assembling Strategy: Formulation Control and Tunable Permeability

**DOI:** 10.3390/nano7110387

**Published:** 2017-11-13

**Authors:** Junbo Li, Lijuan Liang, Ju Liang, Wenlan Wu, Huiyun Zhou, Jinwu Guo

**Affiliations:** 1 School of Chemical Engineering & Pharmaceutics, Henan University of Science & Technology, 263# Kaiyuan Road, Luoyang 471023, China; Lianglijuan@haust.edu.cn (L.L.); Liangju@haust.edu.cn (J.L.); hyzhou@haust.edu.cn (H.Z.); gjinwu@126.com (J.G.); 2 School of Medicine, Henan University of Science & Technology, 263# Kaiyuan Road, Luoyang 471023, China; whenas@sian.com

**Keywords:** asymmetric polymersome, polyion complex, block copolymer, gold template, permeability

## Abstract

A strategy for constructing polyion complex vesicles (PICsomes) with asymmetric structure is described. Poly(methylacrylic acid)-*block*-poly(*N*-isopropylacrylamide) modified gold nanoparticles (PMAA-*b*-PNIPAm-@-Au NPs) were prepared and then assembled with poly(ethylene glycol)-*block*-poly[1-methyl-3-(2-methacryloyloxy propylimidazolium bromine)] (PEG-*b*-PMMPImB) via polyion complex of PMMA and PMMPImB. After removing the Au NPs template, asymmetric PICsomes composed of a PNIPAm inner-shell, PIC wall, and PEG outer-corona were obtained. These PICsomes have low protein absorption and thermally tunable permeability, provided by the PEG outer-corona and the PNIPAm inner-shell, respectively. Moreover, PICsome size can be tailored by using templates of predetermined sizes. This novel strategy for constructing asymmetric PICsomes with well-defined properties and controllable size is valuable for applications such as drug delivery, catalysis and monitoring of chemical reactions, and biomimetics.

## 1. Introduction

Synthetic polymeric vesicles (polymersomes) structuraly based on liposomes [1] have received extensive attention due to their superior stability, adjustable function, and versatile applications [2,3]. The differential functionalization of the outer-corona, middle-wall, and inner-shell [4,5] of asymmetric polymersomes makes them attractive candidates as nanoreactors vessels [6], drug carriers [7], and artificial organelles [8]. For instance, asymmetric polymersomes made with a triblock of copolymers, poly(ethyleneglycol)-*b*-poly(2,4,6-trimethoxybenzylidene-1,1,1-tris (hydroxymethyl) ethane methacrylate)-*b*-poly(-acrylic acid) (PEG-*b*-PTTMA-*b*-PAA), showed biocompatible and “stealthed” properties, efficient drug loading, and acid-tunable permeability [9]. These properties are provided by the PEG outer corona, PAA inner shell, and PTTMA middle membrane, respectively. Most asymmetric polymersomes are synthesized by assembling ABC triblock copolymers [10,11], or by coassembling AB and AC block copolymer (BCP) [4] in selective solvent (where A and C are hydrophilic and B is hydrophobic). However, the synthesis of ABC triblock copolymers is extremely laborious and requires precise control of each block’s chain size. Moreover, poor permeability of the hydrophobic wall and difficult structure identification also limit the applications of these asymmetric polymersomes [12,13]. Thus, a convenient way to prepare asymmetrical polymersomes with a defined structure is still needed [14].

When compared to the assembly of amphipathic BCP, the polyion complex (PIC) approach presents various advantages for preparing polymeric hollow nanomaterials. First, the self-assembled materials are relatively easy to access, as homopolymers, biomacromolecules, and double hydrophlic block copolymers can serve as building blocks, as long as they have opposite charges [6]. Moreover, electrostatic deposition of oppositely charged polyelectrolytes on the surface of the template [15,16,17,18] can be used to control wall thickness and capsule size [6]. Second, PICsomes can directly encapsulate therapeutic biomacromolecules and small molecules by vortex mixing in aqueous medium, and this prepapration process is straightforward and harmless [13,19]. Finally, the PICsome vesicle wall is a semi-permeable membrane that permits the passage of small molecules and ions [13,20,21,22]. Despite these advantages, to widen the applications of PICsomes it will be necessary to develop asymmetric PICsomes with multiple functions.

**Figure 1:**
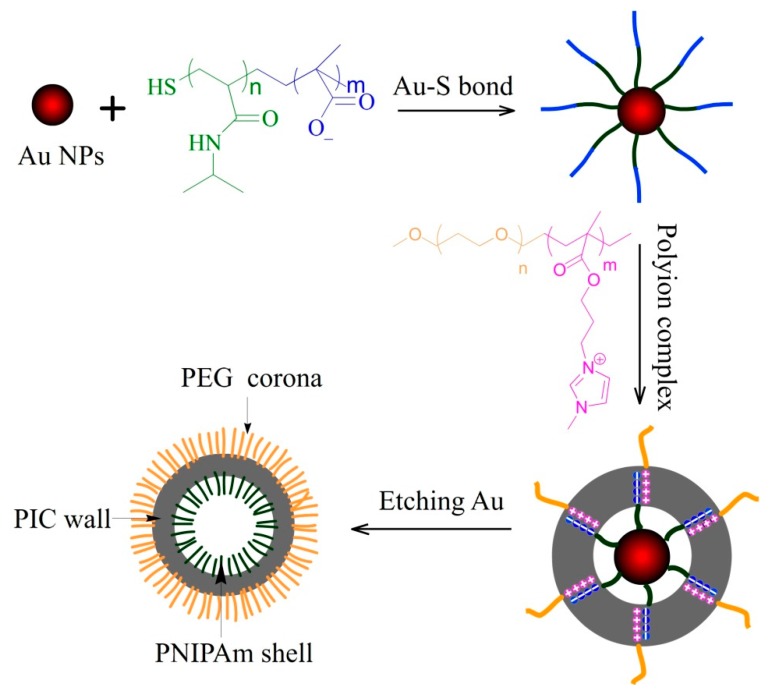
Schematic representation of the synthetic process of asymmetric PICsomes.

Here, we introduce a novel strategy to fabricate asymmetric PICsomes using two double hydrophlic block copolymers (DHBC) with opposite charges, poly(*N*-isopropylacrylamide)-*block*-poly(methylacrylic acid) (PNIPAm-*b*-PMAA) and poly(ethylene glycol)-*block*-poly[1-methyl-3-(2-methacryloyloxy propylimidazolium bromine)] (PEG-*b*-PMMPImB), and gold nanoparticles (Au NPs) as template (Figure 1). PNIPAm-*b*-PMAA was conjugated onto the surface of Au NPs (PMAA-*b*-PNIPAm-@-Au NPs) via formation of Au-S covalent bonds at the chain terminal of PNIPAm. Next, the outer shell of negatively charged PMAA formed an electrostatic complex with the positively charged PMMPImB of PEG-*b*-PMMPImB. After etching gold cores, the PNIPAm segment was encapsulated into the PIC’s wall and PEG was stretched outside. We show that this approach successfully produces PICsomes with asymmetric structure and we analyze the functions provided by their surfaces.

## 2. Experimental Section

### 2.1. Materials 

HAuCl_4_·3H_2_O, sodium citrate, trifluoroacetic acid (TFA) and KCN were purchased from National Pharmaceutical Group Chemical Reagent (Shanghai, China) and used as received. Poly(*N*-isopropylacrylamide)-*block*-poly(methylacrylic acid) with a thiol terminal group (SH-PNIPAm_40_-*b*-PMAA_60_) was prepared through the hydrolysis of PNIPAm-*b*-Pt-BMA with TFA in dry CHCl_3_. Poly(ethylene glycol)-*block*-poly[1-methyl-3-(2-methacryloyloxy propylimidazolium bromine)] (PEG_113_-*b*-PMMPImB_28_) was synthesized via RAFT polymerization by using PEG_113_-CTA as chain trasfer agent and 1-methyl 3-(2-methacryloyloxy propylimidazolium bromine (MMPImB) as monomer. The polydispersity indexes of SH-PNIPAm_40_-*b*-PMAA_60_ and PEG_113_-*b*-PMMPImB_28_ are 1.34 and 1.22, respectively. The detailed synthesis and characterization of SH-PNIPAm-*b*-PMAA and PEG-*b*-PMMPImB were described previously [23,24].

### 2.2. Preparation of PMAA-b-PNIPAm Capped Au NPs

Au NPs of different sizes were first prepared via citrate reduction following an established protocol [25]. SH-PNIPAm_40_-*b*-PMAA_60_ (5 mg) was added to a 20 nm Au NPs (Au_20_ NPs) solution (10 mL, 0.01 wt %) and stirred overnight. The mixture was centrifuged for 5 min at 12,000 rpm, and then the deposition was washed three times to remove excessive polymer. The solid phase of PMAA-*b*-PNIPAm-capped Au_20_ NPs (PMAA_60_-*b*-PNIPAm_40_-@-Au_20_ NPs) was obtained by vacuum drying at 60 °C and was then further characterized by thermogravimetric analysis for determining the content of PMAA-*b*-PNIPAm.

### 2.3. Polyion Complex PMAA-b-PNIPAm-@-Au NPs with PEG-b-PMMPImB

0.1 mg PMAA_60_-*b*-PNIPAm_40_-@-Au_20_ NPs were first dissolved in milliq water to a concentration of 0.05 wt %. Subsequently, a 0.1 wt % PEG_114_-*b*-PMMPImB_28_ aqueous solution was added to the above solution (ca. 10 mL) at a rate of 1 drop every 7 s under vigorous stirring, until the charge ratio of PMAA to PMMPImB was 1. The resulting solution was stirred for 12 h to ensure stable complex formation onto the Au NPs template (PICs-@-Au_20_ NPs). Finally, PICs-@-Au_20_ NPs were purified by three cycles of centrifugation and redispersion. PICs-@-Au NPs made with Au templates of different sizes were prepared following this protocol.

### 2.4. Preparation of Asymmetric PICsomes

100 μL of a stock KCN solution (0.1 g/mL) was added to 10 mL of the PICs-@-Au_20_ NPs solution while stirring. This process was monitored by UV spectroscopy and stopped when the plasmon absorption band disappeared completely. The solution was then immediately dialyzed against water for 24 h to remove small molecular ions. Finally, the polymersome solutions were concentrated by evaporation at room temperature (RT) for further analysis.

### 2.5. Characterization

Dynamic laser scattering (DLS) measurements were performed using a laser light scattering spectrometer (BI-200SM, Brookhaven, New York, NY, USA) equipped with a digital correlator (BI-9000AT, Brookhaven, New York, NY, USA) at 532 nm at RT. Transmission electron microscopy (TEM) measurements were conducted using a JEM-2100 electron microscope (JEOL, Tokyo, Japan) at an acceleration voltage of 200 kV. A small drop of solution was deposited onto a carbon-coated copper EM grid and dried at RT and atmospheric pressure. The UV-Vis spectra were recorded on a Cary 50 Bio UV-Visible Spectrophotometer (Varian, Palo Alto, CA, USA) equipped with two silicon diode detectors and a xenon flash lamp. Atomic force microscope (AFM) measurements were performed using a Nano Wizard^®^II NanoScience AFM (JPK Instruments Inc., Berlin, Germany) at a tapping mode. The diluted solutin of polymersomes were dispensed onto the rinsed glass cover slips and dried at room temperature for 24 h. Zeta potentials were measured using a temperature-controlled Zetasizer 2000 (Malvern Instruments Ltd., Malvern, UK).

## 3. Results and Discussion

### 3.1. Characterization of PICs-@-Au NPs

**Figure 2:**
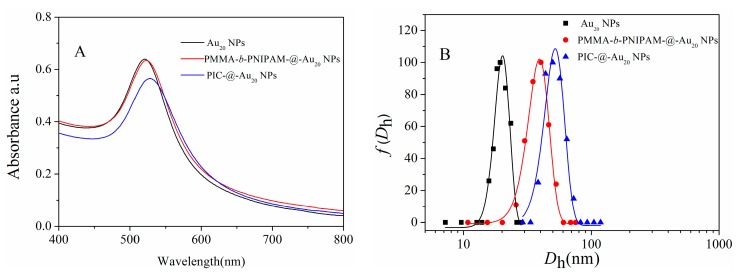

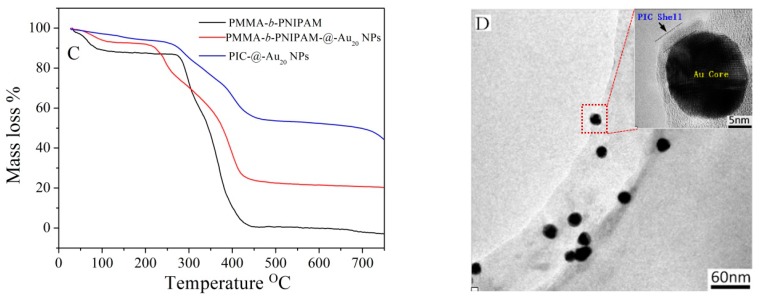
UV-Vis spectra (**A**) and hydrodynamic diameter distributions (**B**) of Au_20_ NPs, PMAA-*b*-PNIPAm-@-Au_20_ NPs and PIC-@-Au_20_ NPs. TGA analysis of PMAA-*b*-PNIPAm, PMAA-*b*-PNIPAm-@-Au_20_ and PIC-@-Au_20_ NPs (**C**). TEM image of PIC-@-Au_20_ NPs (**D**); the inserted image is a magnification of the NPs in (**D**).

We developed a method for preparing asymmetric PICsomes in three main steps: (1) PNIPAm-*b*-PMAA was conjugated to Au NPs via formation of Au-S covalent bonds at the PNIPAm chain terminal; (2) PEG-*b*-PMMPImB was assembled through electrostatic interactions between PMAA negative charges and PMMPImB positive charges; (3) PNIPAm was encapsulated into the PIC’s wall by etching the gold cores and PEG was stretched outside (Figure 1). Polymeric conjugation and PICs formation on the naked surface of Au NPs can be detected by UV-Vis spectroscopy through an increase of refractive index and a red shift in the absorbance spectra [26]. Consistent with this, upon conjugation with PNIPAm-*b*-PMAA, the characteristic surface plasmon resonance (SPR) band of Au_20_ NPs shifted from 521 nm to 523 nm (Figure 2A). Moreover, as PICs formed on the surface of Au NPs, the characteristic SPR band showed a further red shift of 8 nm (to 529 nm), due to an increase of refractive index [26]. The hydrodynamic diameter (*D*_h_) of three nanoparticles was also measured by DLS (Figure 2B). PMAA-*b*-PNIPAm-@-Au_20_ NPs and PIC-@-Au_20_ NPs showed a significantly increased average *D*_h_ from 20 nm to 38 nm and 50 nm. These results are indicative of a successful polymer conjunction and PIC layer formation on the Au NPs surface.

PICs-@-Au NPs characterization by thermogravimetric analysis (TGA) and TEM can further provide information on the core-shell structures. The PMAA-*b*-PNIPAm was found to decompose in temperatures ranging from 276 °C to 480 °C (Figure 2C), corresponding to a 24% content in hybrid NPs. The mean number of grafted polymers was estimated at approximately 1660, and mean density was in the order of 1.31 chains/nm^2^ on each Au_20_ NPs surface, as calculated following a method we described previously [27]. In PIC-@-Au_20_ NPs, the polymer content of each layer was further increased to 54%, and the average number of PEG-*b*-PMMPImB was 1617. According to the results of TGA, the charge ratio of PMAA to PMMPImB is calculated as 0.45, indicating excess negative charges left on the particle surface. The reason can be explained that PMAA-*b*-PNIPAm was anchored to the Au particle surface instead of free chain. The limited conformation of PMAA-*b*-PNIPAm prevents PEG-*b*-PMMPImB from entering deeper shell to fully assemble with PMAA. The zeta potentials of PIC-@-Au NPs is measured and demonstrated PEG-b-PMMPImB is difficult to continue neutralizing negative charges of PMAA when the ratio is over 0.4 (Figure S1). PIC-@-Au_20_ NPs were further characterized by TEM (Figure 2D). The Au NPs core had a uniform size of 20 nm, which is consistent with the size of citrate-reducing Au NPs (Figure S2A). The PIC layer deposited on the Au core had a thickness of approximately 5 nm in dry conditions (magnified single NPs in Figure 2D), which was thicker than PMAA-*b*-PNIPAm-@-Au_20_ NPs (Figure S2B).

### 3.2. Formation of Asymmetric PICsomes

**Figure 3:**
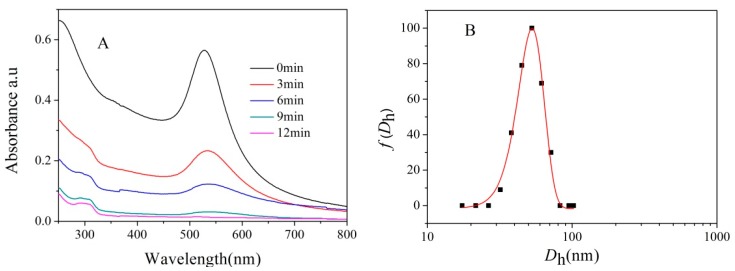

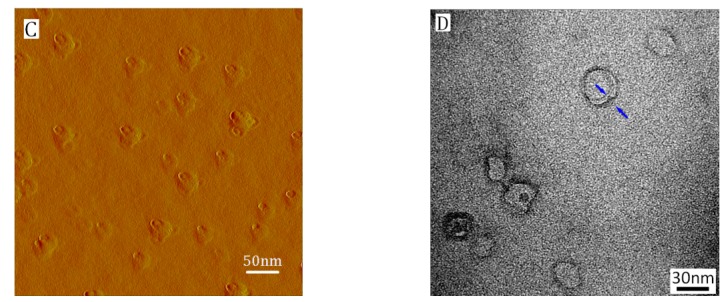
Absorption spectrum of PIC-@-Au_20_ NPs throughout time upon addition of 0.1 g/mL KCN (**A**). Hydrodynamic diameter distributions (**B**). AFM image (**C**), and TEM image (**D**) of PICsomes_20_.

UV-Vis spectroscopy revealed that the SPR absorption of gold (529 nm) gradually disappeared during the last step of synthesis (Figure 3A), demonstrating that the gold core was etched by cyanide. This etching process was rapid (<12 min), and the solution became completely colorless. After dialysis to remove gold cyanide complexes [12], the solution was analyzed with DLS. The detected average *D*_h_ of 52 nm (Figure 3B) revealed the presence of nanoparticles. The morphology of these particles was characterized by atomic force microscope (AFM) and TEM. Spherical aggregates with uniform dimension and a collapsed central structure (height profile, Figure S3) indicate that a hollow structure was formed (Figure 3C) [28]. The smaller size of the nanoparticles, at approximately 45 nm, is due to the collapsed polymer chain in dry conditions. TEM images show that the nanostructure had a clear darker thin wall and hollow lumen (Figure 3D). The hollow nanostructure retains a negatively charged membrane after removing Au core measured by zeta-potential (Figure S4).

Together these results describe a method for constructing PICsomes with an asymmetric structure by prefixing PNIPAm segments on Au NPs and enclosing them in a PIC layer, followed by the random distribution of PEG block on the PIC surface. These particles have a PEG outer-corona, PICs wall, and PNIPAm inner-shell. Importantly, the PEG outer-corona prevents aggregation of PICsomes by steric hindrance.

#### 3.2.1. Size Control of Asymmetric PICsomes

**Figure 4:**
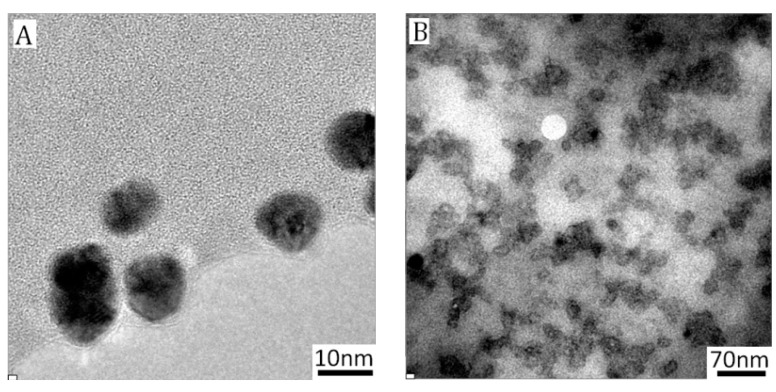

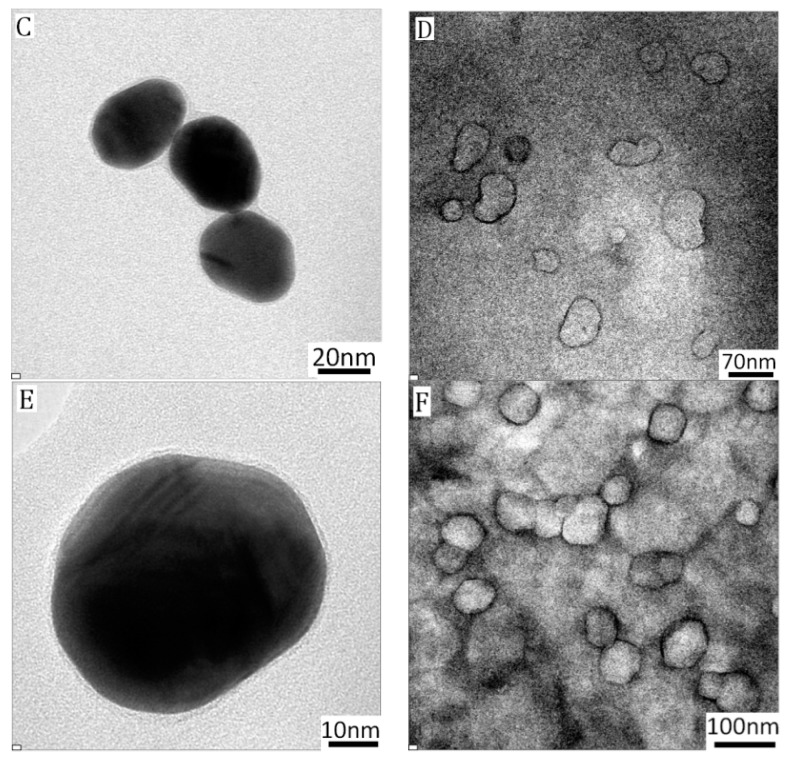
TEM images of PIC-@-Au_10_ NPs (**A**) and PICsome_10_ (**B**); PIC-@-Au_40_ NPs (**C**) and PICsome_40_ (**D**); PIC-@-Au_60_ NPs (**E**) and PICsome_60_ (**F**). The number in subscript corresponds to the size of the Au template.

In addition to their structural functionalization, polymersomes with a defined size are highly desirable for many application, such as drug delivery or organelle modelling. It has been demonstrated that hollow capsule size can be controlled by adjusting the dimension of the template [29]. We predetermined different sizes of Au templates to investigate their effect on asymmetric PICsomes. These PIC-@-Au NPs were first characterized by TEM. As shown in Figure 4A,C,E, external polymeric layers clearly covered Au cores of sizes 10, 43, and 58 nm, indicating that PIC-@-Au NPs formed. These results are supported by data showing SPR red shifts (Figure S5) and increased average *D*_h_ (Figure S6). After removing the Au core, DLS measurements revealed the presence of nanoparticles with *D*_h_ of 35, 67, and 95 nm, with narrow distribution (Figure S7). Clear darker thin walls and hollow lumens (Figure 4B,D,F) observed in TEM images demonstrate that asymmetric PICsomes formed. Figure 4B suggests that PICsome_10_ (the subscript corresponds to the size of the Au template) had a uniform dimension at approximately 26 nm and narrow size distribution. However, PICsomes_43_ (Au template size 43 m) produced two types pf particle, of approximately 60 nm and over 80 nm (Figure 4D). The smaller type (60 nm) may result from etched PIC-@-Au_40_ NPs, which is consistent with the DLS results, whereas the larger particles (>80 nm) could derive from the fusion of small PICsomes_43_. PICsomes of larger sizes have decreased surface curvature and smaller size ratios of PEG shell to lumen, which promotes collision and fusion during the preparation of TEM samples [30]. Consistent with this, PICsomes_58_ formed a single type of large particle (~89 nm) and produced complex fusions (Figure 4F). By using the same polymer and Au templates of different sizes (10, 20, 43, and 58 nm), we were able to produce asymmetric PICsomes of 35, 52, 67, and 95 nm. Collectively, these results demonstrate that the size of asymmetric PICsomes can be controlled by adjusting the size of the Au template, however, PICsome stability depends on the size ratio of polymer to template.

#### 3.2.2. Thermoresponsive Permeability of Asymmetric PICsomes

**Figure 5:**
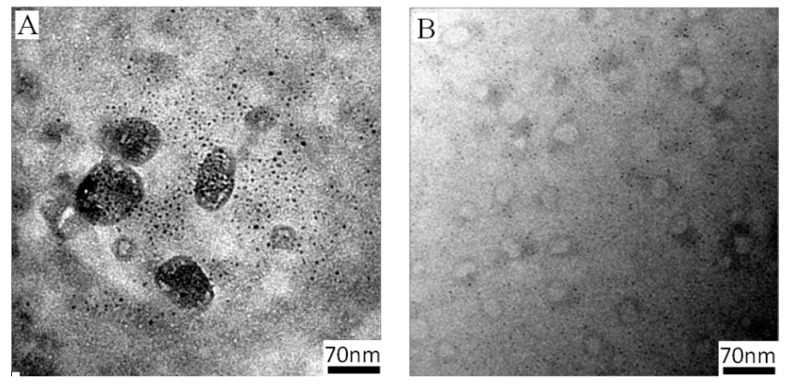
TEM images of PICsome_20_ with 0.1 mg/mL AuCl_4_^−^ at 25 °C (**A**) and 40 °C (**B**).

PNIPAm is a thermo-responsive polymer with the lower critical solution temperature (LCST) around physiological body temperature [31]. For this reason, PNIPAm has been extensively used as a switch for promoting the thermal response of polymersomes, which is extremely useful for controlling drug/molecule release rates [32]. The asymmetric PICsomes fabricated here have a PNIPAm inner-shell and a semi-permeable PIC wall [33] that allows the passage of gold salts at room temperature. To investigate the effects of the PNIPAm’s phase transition (from inner shell to permeability) on PICsome permeability, we compared two PICsome_20_ solutions (10 mL) at either 25 °C or heated to 40 °C over 5 h. After adding 1 mg of HAuCl_4_ to these solutions while stirring, the distribution of AuCl_4_^−^ was observed directly under TEM, as Au NPs form via electron irradiation [34]. The Au NPs that formed at 25 °C were equally distributed inside and outside of PICsomes_20_ (Figure 5A)_._ In contrast, at 40 °C Au NPs were only found outside PICsomes_20_ (Figure 4B), demonstrating that AuCl_4_^−^ cannot enter the lumen at this temperature. Thus, above LCST PNIPAm, forms a hydrophobic membrane and collapses on the wall of PICsomes, resulting in lower permeability. In addition, PICsomes_20_ show good dispersion, and their morphology is unchanged upon phase transition of PNIPAm (Figure 4B). PICsome stability is likely due to their asymmetric structure, as the hydrophlic PEG shell offers excellent surface dispersion properties. Indeed, after incubation with bovine serum albumin (BSA) for 2 h, PICsomes showed only 9% protein adsorption, suggesting the PEG coating provides “stealthed” properties in a physiological environment.

## 4. Conclusions

Here we described a feasible strategy to construct PICsomes with an asymmetric structure. Two DHBC polymers, AB and CD, with opposite charge and a sacrificial template were used. Block A was first anchored on the template surface and then deposited on CD through polyion complex of the B and C blocks. After removing the template, asymmetric PICsomes were produced with the A block as the inner shell, the PIC B with C as middle wall, and the D block as vesicle corona. This methodology allowed us to precisely control the PICsome’s membrane formulation, and to acquire defined functions by using a programmable polymer. Indeed, the asymmetric PICsomes have semi-permeable membranes, biocompatible and “stealthed” properties, and thermally tunable permeability. These properties were provided by the PNIPAm inner-shell, PEG outer-corona, and PIC wall, respectively. Moreover, the size of PICsomes can be controlled by adjusting the size of the template. Thus, we have developed a valuable strategy for engineering asymmetric PICsomes with well-defined size and functions. Given the wide applicability of such particles, this work opens the way for the development and improvement of “smart” drug carriers, nanoreactors, and artificial organelles.

## Supplementary Materials

The following are available online at www.mdpi.com/2079-4991/7/11/387/s1.
