# Supplementary material for: Constructing Asymmetric Polyion Complex Vesicles via Template Assembling Strategy: Formulation Control and Tunable Permeability
Source: Nanomaterials (Basel). 2017 Nov 13;7(11):387. doi: 10.3390/nano7110387 (PMC5707604; doi:10.3390/nano7110387)
Supplement: Supplementary file 1 [file nanomaterials-07-00387-s001.pdf]

Supporting Information

Constructing Asymmetric Polyion Complex Vesicles via Template Assembling Strategy: Formulation Control and Tunable Permeability

Junbo Li [1,*], Lijuan Liang [1], Ju Liang [1], Wenlan Wu [2], Huiyun Zhou [1], and Jinwu Guo [1]

[1] School of Chemical Engineering & Pharmaceutics, Henan University of Science & Technology, 263# Kaiyuan Road, Luo Yang 471023, China; Lianglijuan@haust.edu.cn (L.L.); Liangju@haust.edu.cn (J.L.); hyzhou@haust.edu.cn (H.Z.); gjinwu@126.com (J.G.)

[2] School of Medicine, Henan University of Science & Technology, 263# Kaiyuan Road, Luo Yang 471023, China; whenas@sian.com

* Correspondence: Lijunbo@haust.edu.cn; Tel.: +86-379-6423-2193

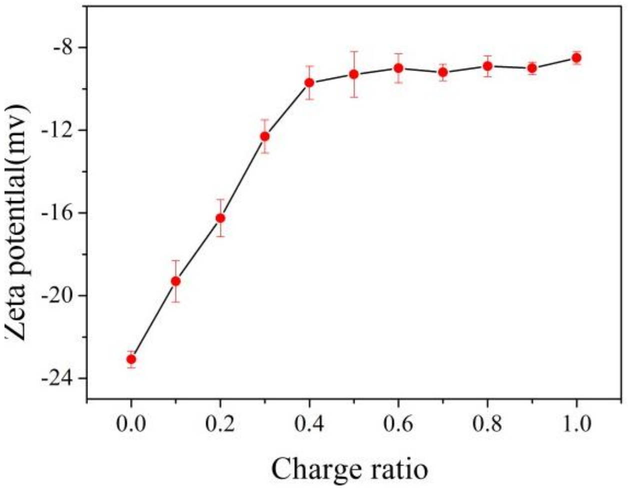

Figure S1. Zeta potentials of PMAA-*b*-PNIPAm-@-Au NPs complex with PEG-*b*-PMMPImB at various charge ratios (PMMA to PMMPImB). Values represent mean (± SD (n = 3))

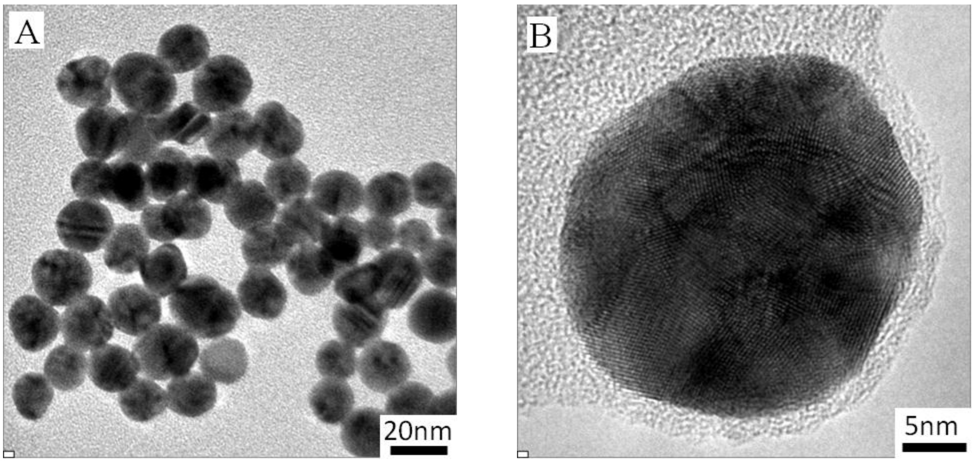

Figure S2. The TEM image of Au 20 NPs(A) and PMAA-*b*-PNIPAm-@-Au_{20} NPs(B)

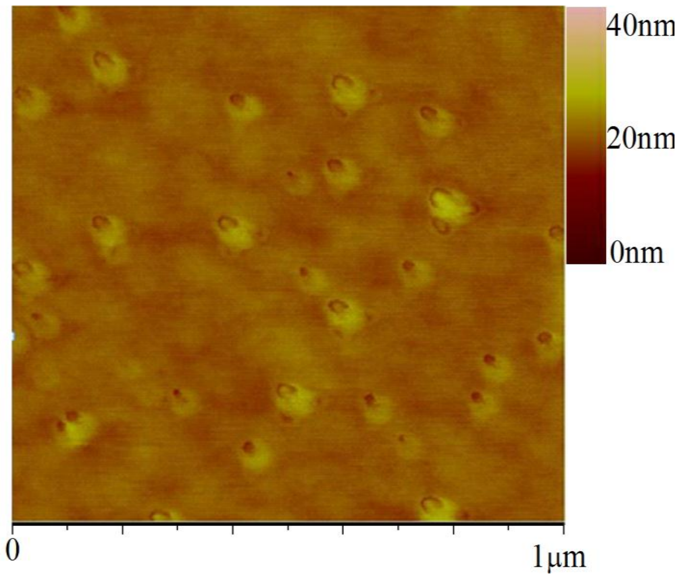

Figure S3. Height profile of PICsomes corresponding to Figure 3C.

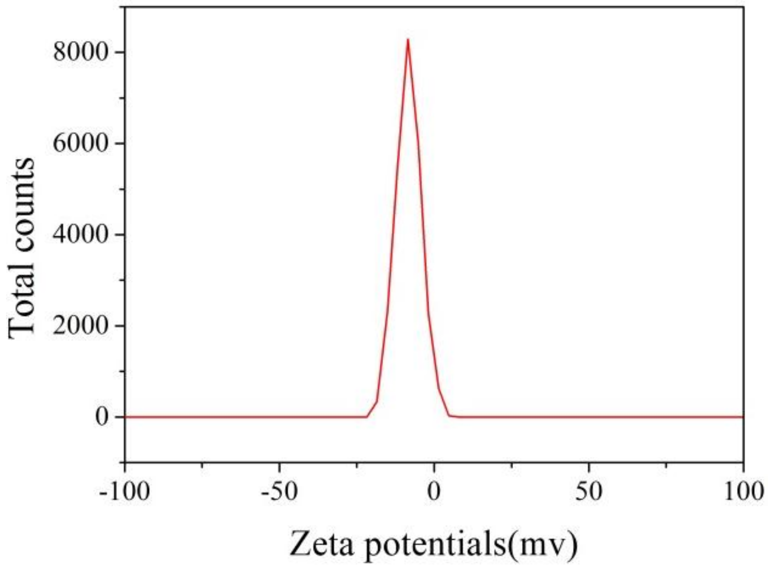

Figure S4. The ζ-potential distribution curves for PICsomes.

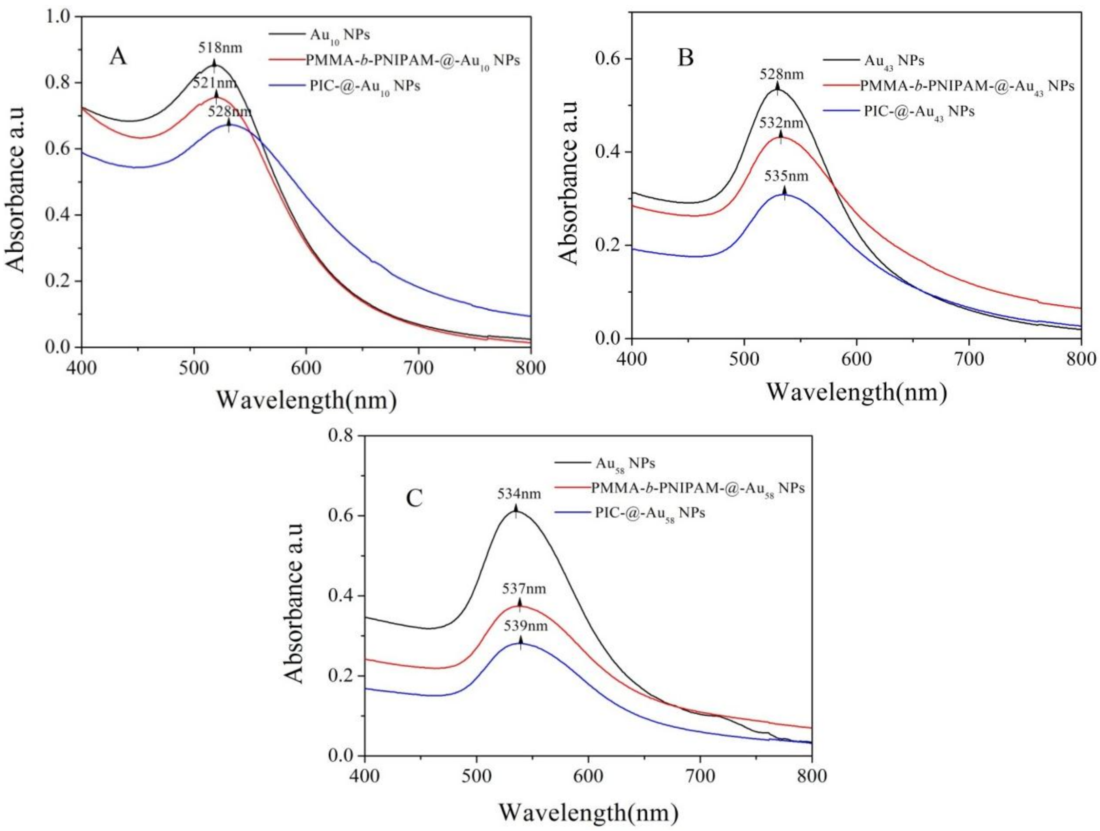

Figure S5. UV-Vis spectra of Au NPs, PMAA-*b*-PNIPAm-@-Au and PIC-@-Au NPs with different size of the Au template, where A is 10nm, B is 43nm and C is 58 nm.

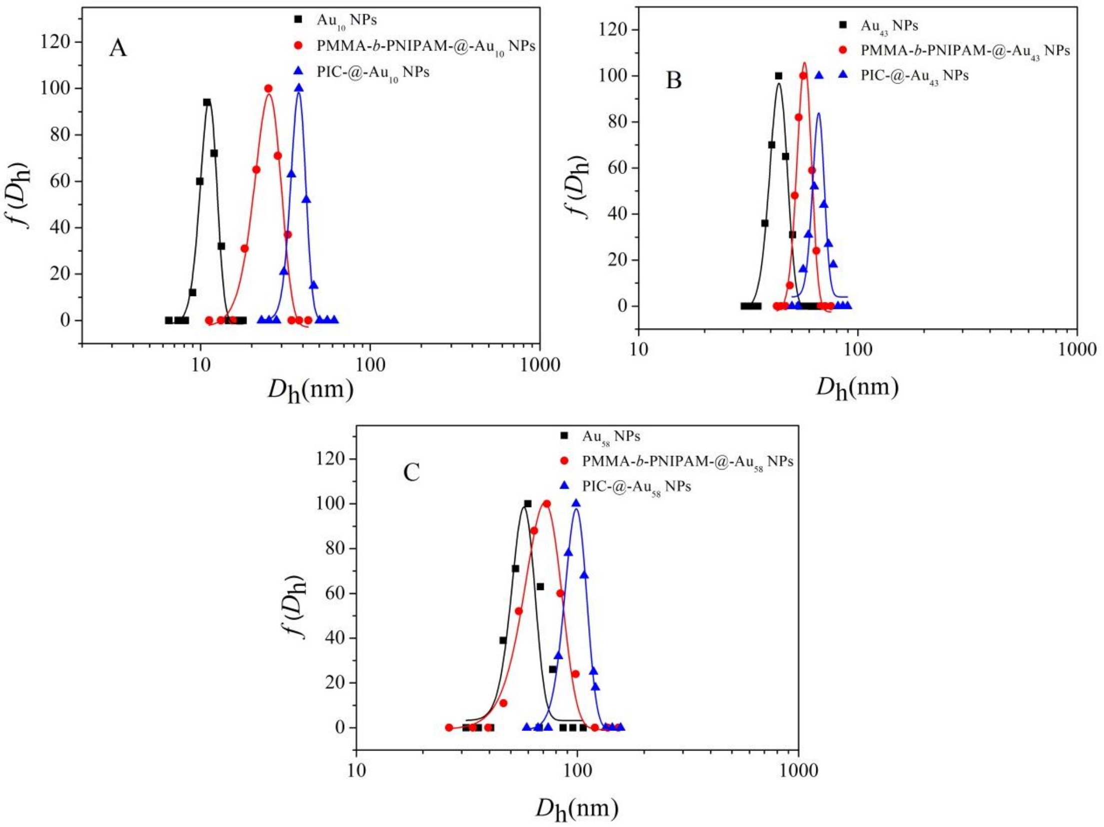

Figure S6. Hydrodynamic diameter distributions of Au NPs, PMAA-*b*-PNIPAm-@-Au and PIC-@-Au NPs with different size of the Au template, where A is 10nm, B is 43nm and C is 58nm.

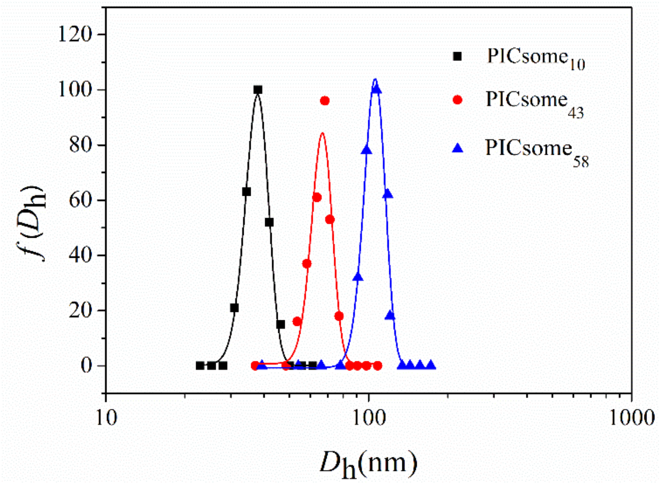

Figure S7. Hydrodynamic diameter distributions of $PICsome_{10}$, $PICsome_{43}$ and $PICsome_{58}$, where the subscript corresponds to the size of the Au template.
